# Supplementary material for: Integrative modelling of reported case numbers and seroprevalence reveals time-dependent test efficiency and infectious contacts
Source: Epidemics. 2023 Jun;43:100681. doi: 10.1016/j.epidem.2023.100681 (PMC10008049; doi:10.1016/j.epidem.2023.100681)
Supplement: MMC S2 — Model description and equations. [file mmc2.pdf]

Model description for "Integrative modelling of reported case numbers and seroprevalence reveals time-dependent test efficiency and infectious contacts"

Symbol	Compartment	Stages for Gamma Chain Trick	Mean transition time
Susc	susceptible individuals	-	(nonlinear)
ExpAsym	exposed individuals who will not develop symptoms	2	$\tau_{\text{exp}\to\text{asym}}$
ExpSym	exposed individuals who will develop symptoms	2	$\tau_{\text{exp}\to\text{sym}}$
Asym	asymptomatic infectious individuals	3	τ_{asym}
ClearAsym	asymptomatic individuals who are no longer infectious	-	(sink state)
Pre	pre-symptomatic infectious individuals	4	τ_{pre}
SymMild	symptomatic individuals who will not require hospitalization	2	$\tau_{\text{recovery,mild}}$
SymSevere	symptomatic individuals who will require hospitalization but no intensive care	2	$\tau_{\text{onset}\to\text{hosp,severe}}$
SymCritical	symptomatic individuals who will require intensive care	2	$\tau_{\text{onset}\to\text{hosp,critical}}$
WardSevere	hospitalized individuals who will not require intensive care	2	$\tau_{\text{recovery,severe}}$
WardCritical	hospitalized individuals who will require intensive care	1	$\tau_{\text{hosp}\to\text{ICU}}$
ICUFatal	individuals in intensive care who will not survive	1	$\tau_{\text{ICU}\to\text{death}}$
ICURecoverable	individuals in intensive care who will survive	1	$\tau_{\text{recovery,critical}}$
RecMild	individuals from SymMild who are no longer symptomatic but still infectious	2	$\tau_{\text{clearance,mild}}$
RecSevere	individuals from SymSevere who are no longer symptomatic but still infectious	2	$\tau_{\text{clearance,severe}}$
RecWard	hospitalized individuals who have recovered from intensive care	1	$\tau_{\text{discharge}}$
ClearSym	symptomatic individuals who are no longer infectious	-	(sink state)
DeadUnreported	deceased individuals who have not yet been reported	1	$1/k_{\text{detect,death}}(t)$
DeadReported	deceased individuals who have been reported	-	(sink state)

Table 1: List of model compartments. To each compartment symbol Comp in the table above, there are actually two associated compartments: Comp, containing only the cases not yet reported to the healthcare authorities, and Comp*, containing only cases that have already been reported. Additionally, a numeric superscript denotes the sub-state generated by the Gamma Chain Trick, such as Comp$^{(k)}$ with $k \in \{1, \ldots, \text{number of sub-states}\}$.

Symbol	Sub-compartments	Description
Exp	ExpAsym ∪ ExpSym	exposed individuals
Clear	ClearSym ∪ ClearAsym	no longer infectious individuals
Sym	SymMild ∪ SymSevere ∪ SymCritical	symptomatic (but not hospitalized) individuals
Ward	WardSevere ∪ WardCritical ∪ RecWard	hospitalized individuals not in the ICU
ICU	ICUFatal ∪ ICURecoverable	individuals in the ICU
Hospitalized	Ward ∪ ICU	hospitalized individuals
Rec	RecMild ∪ RecSevere	non-hospitalized individuals who are no longer symptomatic
Dead	DeadUnreported ∪ DeadReported	deceased individuals

Table 2: List of short-hands for unions of two or more compartments.

Detection rate	Compartments
$k_{\text{detect,asym}}(t)$	Asym, Pre
$k_{\text{detect,sym}}(t)$	Sym
∞	Hospitalized, Dead
0	Exp, Rec, Clear

Table 3: Rates at which cases are reported to the healthcare authorities ("detected"). A detection rate equal to ∞ means that cases are automatically detected on entry to that compartment; as a consequence only the * state associated to that compartment exists. A detection rate of 0 does not preclude the existence of an associated * state, since individuals may have been detected at previous stages.

$\tilde{\beta}$	Compartments
$\tilde{\beta}_{\text{quarantine}}$	all * compartments (detected individuals)
$\tilde{\beta}_{\text{sick}}$	Sym
$\tilde{\beta}_{\text{NPI}}(t) \coloneqq \rho_{\text{NPI}}(t)\,\tilde{\beta}_0 + (1-\rho_{\text{NPI}}(t))\,\tilde{\beta}_{\text{sick}}$	Asym, Pre

Table 4: Average number of potentially infectious contacts $\tilde{\beta}$ that individuals from the various compartments have during one day. The reasonable constraint $\tilde{\beta}_{\text{quarantine}} \leq \tilde{\beta}_{\text{sick}}$ is enforced, while the equally reasonable $\tilde{\beta}_{\text{sick}} \leq \tilde{\beta}_{\text{NPI}}$ holds by construction since ρ_{NPI} is always positive.

γ	Compartments
$\gamma_{\text{asymptomatic}}$	Asym
$\gamma_{\text{presymptomatic}}$	Pre
$\gamma_{\text{symptomatic}}$	Sym, WardSevere, WardCritical
$\gamma_{\text{recovered}}$	Rec
0	Exp, ICU

Table 5: Infectiousness level γ for each model compartment, i.e., the fraction of potentially infectious contacts $\tilde{\beta}$ which actually result in an infection.

Parameter	Description
r	infection rate
$N_0 = 1,561,720$	initial population
$\tilde{\beta}_0 = 7.769$	average number of potentially infectious contacts an individual has in one day in a pre-pandemic setting
$\rho_{\mathrm{NPI}}(t)$	effectiveness of NPIs at a given timepoint<br>(always positive, 1 corresponds to a pre-pandemic situation and 0 to the most effective NPIs)
f_{asym}	fraction of exposed individuals who do not develop symptoms
f_{hosp}	fraction of symptomatic individuals who are hospitalized
f_{ICU}	fraction of hospitalized individuals who require intensive care
m_{ICU}	fraction of individuals in ICU who do not survive
$f_{\mathrm{infected},0}$	fraction of the total population who is infected with the virus at the simulation start
$f_{\mathrm{infectious},0}$	fraction of infected individuals who are infectious at the simulation start
$f_{\mathrm{symptomatic},0}$	fraction of infectious individuals on the symptomatic model branch who have already had symptoms the simulation start

Table 6: Other parameters.

ODEs

$$\frac{\mathrm{d}}{\mathrm{d}t}\mathrm{Susc} = -r\,\mathrm{Susc}$$

$$\frac{\mathrm{d}}{\mathrm{d}t}\mathrm{ExpAsym}^{(1)} = f_{\mathrm{asym}} r\,\mathrm{Susc} - \frac{\mathrm{ExpAsym}^{(1)}}{\tau_{\mathrm{exp}\to\mathrm{asym}}/2}$$

$$\frac{\mathrm{d}}{\mathrm{d}t}\mathrm{ExpAsym}^{(2)} = \frac{\mathrm{ExpAsym}^{(1)} - \mathrm{ExpAsym}^{(2)}}{\tau_{\mathrm{exp}\to\mathrm{asym}}/2}$$

$$\frac{\mathrm{d}}{\mathrm{d}t}\mathrm{ExpSym}^{(1)} = (1 - f_{\mathrm{asym}})\, r\,\mathrm{Susc} - \frac{\mathrm{ExpSym}^{(1)}}{\tau_{\mathrm{exp}\to\mathrm{sym}}/2}$$

$$\frac{\mathrm{d}}{\mathrm{d}t}\mathrm{ExpSym}^{(2)} = \frac{\mathrm{ExpSym}^{(1)} - \mathrm{ExpSym}^{(2)}}{\tau_{\mathrm{exp}\to\mathrm{sym}}/2}$$

$$\frac{\mathrm{d}}{\mathrm{d}t}\mathrm{Asym}^{(1)} = \frac{\mathrm{ExpAsym}^{(2)}}{\tau_{\mathrm{exp}\to\mathrm{asym}}/2} - \frac{\mathrm{Asym}^{(1)}}{\tau_{\mathrm{asym}}/3} - k_{\mathrm{detect,asym}}\mathrm{Asym}^{(1)}$$

$$\frac{\mathrm{d}}{\mathrm{d}t}\mathrm{Asym}^{*(1)} = -\frac{\mathrm{Asym}^{*(1)}}{\tau_{\mathrm{asym}}/3} + k_{\mathrm{detect,asym}}\mathrm{Asym}^{(1)}$$

$$\frac{\mathrm{d}}{\mathrm{d}t}\mathrm{Asym}^{(2)} = \frac{\mathrm{Asym}^{(1)} - \mathrm{Asym}^{(2)}}{\tau_{\mathrm{asym}}/3} - k_{\mathrm{detect,asym}}\mathrm{Asym}^{(2)}$$

$$\frac{\mathrm{d}}{\mathrm{d}t}\mathrm{Asym}^{*(2)} = \frac{\mathrm{Asym}^{*(1)} - \mathrm{Asym}^{*(2)}}{\tau_{\mathrm{asym}}/3} + k_{\mathrm{detect,asym}}\mathrm{Asym}^{(2)}$$

$$\frac{\mathrm{d}}{\mathrm{d}t}\mathrm{Asym}^{(3)} = \frac{\mathrm{Asym}^{(2)} - \mathrm{Asym}^{(3)}}{\tau_{\mathrm{asym}}/3} - k_{\mathrm{detect,asym}}\mathrm{Asym}^{(3)}$$

$$\frac{\mathrm{d}}{\mathrm{d}t}\mathrm{Asym}^{*(3)} = \frac{\mathrm{Asym}^{*(2)} - \mathrm{Asym}^{*(3)}}{\tau_{\mathrm{asym}}/3} + k_{\mathrm{detect,asym}}\mathrm{Asym}^{(3)}$$

$$\frac{\mathrm{d}}{\mathrm{d}t}\mathrm{ClearAsym} = \frac{\mathrm{Asym}^{(3)}}{\tau_{\mathrm{asym}}/3}$$

$$\frac{\mathrm{d}}{\mathrm{d}t}\mathrm{ClearAsym}^{*} = \frac{\mathrm{Asym}^{*(3)}}{\tau_{\mathrm{asym}}/3}$$

$$\frac{\mathrm{d}}{\mathrm{d}t}\mathrm{Pre}^{(1)} = \frac{\mathrm{ExpSym}^{(2)}}{\tau_{\mathrm{exp}\to\mathrm{sym}}/2} - \frac{\mathrm{Pre}^{(1)}}{\tau_{\mathrm{pre}}/4} - k_{\mathrm{detect,asym}}\mathrm{Pre}^{(1)}$$

$$\frac{\mathrm{d}}{\mathrm{d}t}\mathrm{Pre}^{*(1)} = -\frac{\mathrm{Pre}^{*(1)}}{\tau_{\mathrm{pre}}/4} + k_{\mathrm{detect,asym}}\mathrm{Pre}^{(1)}$$

$$\frac{\mathrm{d}}{\mathrm{d}t}\mathrm{Pre}^{(2)} = \frac{\mathrm{Pre}^{(1)} - \mathrm{Pre}^{(2)}}{\tau_{\mathrm{pre}}/4} - k_{\mathrm{detect,asym}}\mathrm{Pre}^{(2)}$$

$$\frac{\mathrm{d}}{\mathrm{d}t}\mathrm{Pre}^{*(2)} = \frac{\mathrm{Pre}^{*(1)} - \mathrm{Pre}^{*(2)}}{\tau_{\mathrm{pre}}/4} + k_{\mathrm{detect,asym}}\mathrm{Pre}^{(2)}$$

$$\frac{\mathrm{d}}{\mathrm{d}t}\mathrm{Pre}^{(3)} = \frac{\mathrm{Pre}^{(2)} - \mathrm{Pre}^{(3)}}{\tau_{\mathrm{pre}}/4} - k_{\mathrm{detect,asym}}\mathrm{Pre}^{(3)}$$

$$\frac{\mathrm{d}}{\mathrm{d}t}\mathrm{Pre}^{*(3)} = \frac{\mathrm{Pre}^{*(2)} - \mathrm{Pre}^{*(3)}}{\tau_{\mathrm{pre}}/4} + k_{\mathrm{detect,asym}}\mathrm{Pre}^{(3)}$$

$$\frac{\mathrm{d}}{\mathrm{d}t}\mathrm{Pre}^{(4)} = \frac{\mathrm{Pre}^{(3)} - \mathrm{Pre}^{(4)}}{\tau_{\mathrm{pre}}/4} - k_{\mathrm{detect,asym}}\mathrm{Pre}^{(4)}$$

$$\frac{\mathrm{d}}{\mathrm{d}t}\mathrm{Pre}^{*(4)} = \frac{\mathrm{Pre}^{*(3)} - \mathrm{Pre}^{*(4)}}{\tau_{\mathrm{pre}}/4} + k_{\mathrm{detect,asym}}\mathrm{Pre}^{(4)}$$

$$\frac{\mathrm{d}}{\mathrm{d}t}\mathrm{SymMild}^{(1)} = (1 - f_{\mathrm{hosp}})\frac{\mathrm{Pre}^{(4)}}{\tau_{\mathrm{pre}}/4} - \frac{\mathrm{SymMild}^{(1)}}{\tau_{\mathrm{recovery,mild}}/2} - k_{\mathrm{detect,sym}}\mathrm{SymMild}^{(1)}$$

$$\frac{\mathrm{d}}{\mathrm{d}t}\mathrm{SymMild}^{*(1)} = (1 - f_{\mathrm{hosp}})\frac{\mathrm{Pre}^{*(4)}}{\tau_{\mathrm{pre}}/4} - \frac{\mathrm{SymMild}^{*(1)}}{\tau_{\mathrm{recovery,mild}}/2} + k_{\mathrm{detect,sym}}\mathrm{SymMild}^{(1)}$$

$$\frac{\mathrm{d}}{\mathrm{d}t}\mathrm{SymMild}^{(2)} = \frac{\mathrm{SymMild}^{(1)} - \mathrm{SymMild}^{(2)}}{\tau_{\mathrm{recovery,mild}}/2} - k_{\mathrm{detect,sym}}\mathrm{SymMild}^{(2)}$$

$$\frac{\mathrm{d}}{\mathrm{d}t}\mathrm{SymMild}^{*(2)} = \frac{\mathrm{SymMild}^{*(1)} - \mathrm{SymMild}^{*(2)}}{\tau_{\mathrm{recovery,mild}}/2} + k_{\mathrm{detect,sym}}\mathrm{SymMild}^{(2)}$$

$$\frac{\mathrm{d}}{\mathrm{d}t}\mathrm{SymSevere} = (1 - f_{\mathrm{ICU}})\, f_{\mathrm{hosp}}\frac{\mathrm{Pre}^{(4)}}{\tau_{\mathrm{pre}}/4} - \frac{\mathrm{SymSevere}}{\tau_{\mathrm{onset}\to\mathrm{hosp,severe}}} - k_{\mathrm{detect,sym}}\mathrm{SymSevere}$$

$$\frac{\mathrm{d}}{\mathrm{d}t}\mathrm{SymSevere}^{*} = (1 - f_{\mathrm{ICU}})\, f_{\mathrm{hosp}}\frac{\mathrm{Pre}^{*(4)}}{\tau_{\mathrm{pre}}/4} - \frac{\mathrm{SymSevere}^{*}}{\tau_{\mathrm{onset}\to\mathrm{hosp,severe}}} + k_{\mathrm{detect,sym}}\mathrm{SymSevere}$$

$$\frac{\mathrm{d}}{\mathrm{d}t}\mathrm{SymCritical}^{(1)} = f_{\mathrm{ICU}} f_{\mathrm{hosp}}\frac{\mathrm{Pre}^{(4)}}{\tau_{\mathrm{pre}}/4} - \frac{\mathrm{SymCritical}^{(1)}}{\tau_{\mathrm{onset}\to\mathrm{hosp,critical}}/2} - k_{\mathrm{detect,sym}}\mathrm{SymCritical}^{(1)}$$

$$\frac{\mathrm{d}}{\mathrm{d}t}\mathrm{SymCritical}^{*(1)} = f_{\mathrm{ICU}} f_{\mathrm{hosp}}\frac{\mathrm{Pre}^{*(4)}}{\tau_{\mathrm{pre}}/4} - \frac{\mathrm{SymCritical}^{*(1)}}{\tau_{\mathrm{onset}\to\mathrm{hosp,critical}}/2} + k_{\mathrm{detect,sym}}\mathrm{SymCritical}^{(1)}$$

$$\frac{\mathrm{d}}{\mathrm{d}t}\mathrm{SymCritical}^{(2)} = \frac{\mathrm{SymCritical}^{(1)} - \mathrm{SymCritical}^{(2)}}{\tau_{\mathrm{onset}\to\mathrm{hosp,critical}}/2} - k_{\mathrm{detect,sym}}\mathrm{SymCritical}^{(2)}$$

$$\frac{\mathrm{d}}{\mathrm{d}t}\mathrm{SymCritical}^{*(2)} = \frac{\mathrm{SymCritical}^{*(1)} - \mathrm{SymCritical}^{*(2)}}{\tau_{\mathrm{onset}\to\mathrm{hosp,critical}}/2} + k_{\mathrm{detect,sym}}\mathrm{SymCritical}^{(2)}$$

$$\frac{\mathrm{d}}{\mathrm{d}t}\mathrm{WardSevere}^{*(1)} = \frac{\mathrm{SymSevere} + \mathrm{SymSevere}^{*}}{\tau_{\mathrm{onset}\to\mathrm{hosp,severe}}} - \frac{\mathrm{WardSevere}^{*(1)}}{\tau_{\mathrm{ward}\to\mathrm{recovered}}/2}$$

$$\frac{\mathrm{d}}{\mathrm{d}t}\mathrm{WardSevere}^{*(2)} = \frac{\mathrm{WardSevere}^{*(1)} - \mathrm{WardSevere}^{*(2)}}{\tau_{\mathrm{ward}\to\mathrm{recovered}}/2}$$

$$\frac{\mathrm{d}}{\mathrm{d}t}\mathrm{WardCritical}^{*} = \frac{\mathrm{SymCritical}^{(2)} + \mathrm{SymCritical}^{*(2)}}{\tau_{\mathrm{onset}\to\mathrm{hosp,critical}}/2} - \frac{\mathrm{WardCritical}^{*}}{\tau_{\mathrm{hosp}\to\mathrm{ICU}}}$$

$$\frac{\mathrm{d}}{\mathrm{d}t}\mathrm{ICUFatal}^{*} = m_{\mathrm{ICU}}\frac{\mathrm{WardCritical}^{*}}{\tau_{\mathrm{hosp}\to\mathrm{ICU}}} - \frac{\mathrm{ICUFatal}^{*}}{\tau_{\mathrm{ICU}\to\mathrm{death}}}$$

$$\frac{\mathrm{d}}{\mathrm{d}t}\mathrm{ICURecoverable}^{*} = (1 - m_{\mathrm{ICU}})\frac{\mathrm{WardCritical}^{*}}{\tau_{\mathrm{hosp}\to\mathrm{ICU}}} - \frac{\mathrm{ICURecoverable}^{*}}{\tau_{\mathrm{recovery,critical}}}$$

$$\frac{\mathrm{d}}{\mathrm{d}t}\mathrm{RecMild}^{(1)} = \frac{\mathrm{SymMild}^{(2)}}{\tau_{\mathrm{recovery,mild}}/2} - \frac{\mathrm{RecMild}^{(1)}}{\tau_{\mathrm{clearance,mild}}/2}$$

$$\frac{\mathrm{d}}{\mathrm{d}t}\mathrm{RecMild}^{*(1)} = \frac{\mathrm{SymMild}^{*(2)}}{\tau_{\mathrm{recovery,mild}}/2} - \frac{\mathrm{RecMild}^{*(1)}}{\tau_{\mathrm{clearance,mild}}/2}$$

$$\frac{\mathrm{d}}{\mathrm{d}t}\mathrm{RecMild}^{(2)} = \frac{\mathrm{RecMild}^{(1)} - \mathrm{RecMild}^{(2)}}{\tau_{\mathrm{clearance,mild}}/2}$$

$$\frac{\mathrm{d}}{\mathrm{d}t}\mathrm{RecMild}^{*(2)} = \frac{\mathrm{RecMild}^{*(1)} - \mathrm{RecMild}^{*(2)}}{\tau_{\mathrm{clearance,mild}}/2}$$

$$\frac{\mathrm{d}}{\mathrm{d}t}\mathrm{RecSevere}^{*(1)} = \frac{\mathrm{WardSevere}^{*(2)}}{\tau_{\mathrm{ward}\to\mathrm{recovered}}/2} - \frac{\mathrm{RecSevere}^{*(1)}}{\tau_{\mathrm{clearance,severe}}/2}$$

$$\frac{\mathrm{d}}{\mathrm{d}t}\mathrm{RecSevere}^{*(2)} = \frac{\mathrm{RecSevere}^{*(1)} - \mathrm{RecSevere}^{*(2)}}{\tau_{\mathrm{clearance,severe}}/2}$$

$$\frac{\mathrm{d}}{\mathrm{d}t}\mathrm{RecWard}^{*} = \frac{\mathrm{ICURecoverable}^{*}}{\tau_{\mathrm{recovery,critical}}} - \frac{\mathrm{RecWard}^{*}}{\tau_{\mathrm{discharge}}}$$

$$\frac{\mathrm{d}}{\mathrm{d}t}\mathrm{ClearSym} = \frac{\mathrm{RecMild}^{(2)}}{\tau_{\mathrm{clearance,mild}}/2}$$

$$\frac{\mathrm{d}}{\mathrm{d}t}\mathrm{ClearSym}^{*} = \frac{\mathrm{RecMild}^{*(2)}}{\tau_{\mathrm{clearance,mild}}/2} + \frac{\mathrm{RecSevere}^{*(2)}}{\tau_{\mathrm{clearance,severe}}/2} + \frac{\mathrm{RecWard}^{*}}{\tau_{\mathrm{discharge}}}$$

$$\frac{\mathrm{d}}{\mathrm{d}t}\mathrm{DeadUnreported}^{*} = \frac{\mathrm{ICUFatal}^{*}}{\tau_{\mathrm{ICU}\to\mathrm{death}}} - k_{\mathrm{detect,death}}\mathrm{DeadUnreported}^{*}$$

$$\frac{\mathrm{d}}{\mathrm{d}t}\mathrm{DeadReported}^{*} = k_{\mathrm{detect,death}}\mathrm{DeadUnreported}^{*}$$

Infection rate

$$\begin{aligned} N_0 r = \;& \tilde{\beta}_{\mathrm{NPI}}\gamma_{\mathrm{asymptomatic}}\mathrm{Asym} \\ &+ \tilde{\beta}_{\mathrm{NPI}}\gamma_{\mathrm{presymptomatic}}\mathrm{Pre} \\ &+ \tilde{\beta}_{\mathrm{sick}}\gamma_{\mathrm{symptomatic}}\left(\mathrm{SymCritical} + \mathrm{SymMild} + \mathrm{SymSevere}\right) \\ &+ \tilde{\beta}_{\mathrm{NPI}}\gamma_{\mathrm{recovered}}\mathrm{RecMild} \\ &+ \tilde{\beta}_{\mathrm{quarantine}}\gamma_{\mathrm{asymptomatic}}\mathrm{Asym}^{*} \\ &+ \tilde{\beta}_{\mathrm{quarantine}}\gamma_{\mathrm{presymptomatic}}\mathrm{Pre}^{*} \\ &+ \tilde{\beta}_{\mathrm{quarantine}}\gamma_{\mathrm{symptomatic}}\left(\mathrm{SymCritical}^{*} + \mathrm{SymMild}^{*} + \mathrm{SymSevere}^{*} + \mathrm{WardCritical}^{*} + \mathrm{WardSevere}^{*}\right) \\ &+ \tilde{\beta}_{\mathrm{quarantine}}\gamma_{\mathrm{recovered}}\left(\mathrm{RecMild}^{*} + \mathrm{RecSevere}^{*}\right) \end{aligned}$$

Initial conditions

$$\mathrm{Susc}(0) = \left(1 - f_{\mathrm{infected},0}\right) N_0$$

$$\mathrm{ExpAsym}^{(i)}(0) = \frac{1}{2} f_{\mathrm{asym}}\left(1 - f_{\mathrm{infectious},0}\right) f_{\mathrm{infected},0} N_0, \quad i = 1,2$$

$$\mathrm{ExpSym}^{(i)}(0) = \frac{1}{2}\left(1 - f_{\mathrm{asym}}\right)\left(1 - f_{\mathrm{infectious},0}\right) f_{\mathrm{infected},0}\, N_0, \quad i = 1,2$$

$$\mathrm{Asym}^{(i)}(0) = \frac{1}{3} f_{\mathrm{asym}}\, f_{\mathrm{infectious},0}\, f_{\mathrm{infected},0}\, N_0, \quad i = 1,2,3$$

$$\mathrm{Pre}^{(i)}(0) = \frac{1}{4}\left(1 - f_{\mathrm{symptomatic},0}\right)\left(1 - f_{\mathrm{asym}}\right) f_{\mathrm{infectious},0}\, f_{\mathrm{infected},0}\, N_0, \quad i = 1,2,3,4$$

$$\mathrm{SymMild}^{(i)}(0) = \frac{1}{2}\left(1 - f_{\mathrm{hosp}}\right) f_{\mathrm{symptomatic},0}\left(1 - f_{\mathrm{asym}}\right) f_{\mathrm{infectious},0}\, f_{\mathrm{infected},0}\, N_0, \quad i = 1,2$$

$$\mathrm{SymSevere}(0) = \left(1 - f_{\mathrm{ICU}}\right) f_{\mathrm{hosp}}\, f_{\mathrm{symptomatic},0}\left(1 - f_{\mathrm{asym}}\right) f_{\mathrm{infectious},0}\, f_{\mathrm{infected},0}\, N_0$$

$$\mathrm{SymCritical}^{(i)}(0) = \frac{1}{2} f_{\mathrm{ICU}}\, f_{\mathrm{hosp}}\, f_{\mathrm{symptomatic},0}\left(1 - f_{\mathrm{asym}}\right) f_{\mathrm{infectious},0}\, f_{\mathrm{infected},0}\, N_0, \quad i = 1,2$$

$$\text{all other compartments, in particular all }^{*}\text{ compartments} = 0$$

Observables

Rate at which new cases are reported to the healthcare authorities

$$\frac{\mathrm{SymSevere}}{\tau_{\mathrm{onset}\to\mathrm{hosp,severe}}} + \frac{\mathrm{SymCritical}^{(2)}}{\tau_{\mathrm{onset}\to\mathrm{hosp,critical}}/2} + k_{\mathrm{detect,asym}}\left(\mathrm{Asym} + \mathrm{Pre}\right) + k_{\mathrm{detect,sym}}\mathrm{Sym}$$

Rate at which deaths are reported to the healthcare authorities

$$k_{\mathrm{detect,death}}\mathrm{DeadUnreported}^{*}$$

Rate at which symptom onsets are reported to the healthcare authorities (by individuals who have been detected after the onset of symptoms)

$$p_{\mathrm{detected}}\frac{\mathrm{Pre}^{(4)}}{\tau_{\mathrm{pre}}/4}$$

where p_{detected} is the probability that a person who is showing symptoms for the first time and has not been detected yet will be detected before the virus is cleared from their system, as given by the formula

$$p_{\mathrm{detected}} = f_{\mathrm{detected\ onsets,sym}}\left[1 - (1 - f_{\mathrm{hosp}})\left(\frac{\tau_{\mathrm{test,sym,0}}}{\tau_{\mathrm{recovery,mild}}/2 + \tau_{\mathrm{test,sym,0}}}\right)^{2}\right]$$

Rate at which symptom onsets are reported to the healthcare authorities (by individuals who have been detected before the onset of symptoms, i.e., while presymptomatic)

$$f_{\mathrm{detected\ onsets,asym}}\frac{\mathrm{Pre}^{*(4)}}{\tau_{\mathrm{pre}}/4}$$

Number of hospital ward (not ICU) beds occupied by COVID-19 patients

$$f_{\mathrm{observed\ beds,severe}}\mathrm{WardSevere}^{*} + f_{\mathrm{observed\ beds,critical}}\left(\mathrm{RecWard}^{*} + \mathrm{WardCritical}^{*}\right)$$

Number of hospital ICU beds occupied by COVID-19 patients

$$f_{\mathrm{observed\ beds,critical}}\mathrm{ICU}^{*}$$

Measured prevalence of COVID-19 antibodies in the population at time t

$$(\mathrm{se} + \mathrm{sp} - 1)\frac{\hat{N}(t - 2\,\mathrm{weeks})}{N_0} + (1 - \mathrm{sp})$$

where $\mathrm{se} = 0.8860104$ and $\mathrm{sp} = 0.9972041$ are the sensitivity and specificity of the antibody test, while $\hat{N}(t)$ is the number of individuals who will possess COVID-19 antibodies two weeks after the time point t, as given by

$$\begin{aligned}\hat{N} = \quad & \mathrm{Asym} + \mathrm{Asym}^{*} + \mathrm{SymMild} + \mathrm{SymMild}^{*} + \mathrm{SymSevere} + \mathrm{SymSevere}^{*} \\ & + \mathrm{WardSevere}^{*} + \mathrm{ICURecoverable}^{*} + \mathrm{Rec} + \mathrm{Rec}^{*} + \mathrm{RecWard}^{*} + \mathrm{Clear} + \mathrm{Clear}^{*} \\ & + (1 - m_{\mathrm{ICU}})\left(\mathrm{SymCritical} + \mathrm{SymCritical}^{*} + \mathrm{WardCritical}^{*}\right) + (1 - m_{\mathrm{ICU}}f_{\mathrm{ICU}}f_{\mathrm{hosp}})\left(\mathrm{Pre} + \mathrm{Pre}^{*}\right)\end{aligned}$$
